# Review of residential and nursing care home policies on safety incident reporting in England

**DOI:** 10.1177/13558196251392508

**Published:** 2025-11-21

**Authors:** Mel Steer, Kate Sykes, Justin Waring, Celia Mason, Pamela Dawson, Craig Newman, Lesley Young-Murphy, Michele Spencer, Jason Scott

**Affiliations:** 1 School of Healthcare and Nursing Sciences, 373117 Northumbria University, England; 2 School of Communities and Education, 373117 Northumbria University, England; 3 School of Social Sciences, Loughborough University, England; 4 Deputy Vice Chancellor’s Office, 6629 Plymouth Marjon University; 5 North Tyneside Community and Health Care Forum

**Keywords:** incident reporting, safety, care homes

## Abstract

**Objectives:**

In care homes, safety incident reporting, and the policy framework that surrounds safety incident reporting, is not well understood. This study aims to review safety incident reporting and safety policies in residential and nursing care homes in England. It aims to better understand safety incident reporting practices and identify lessons for the sector regarding approaches to safety incident reporting to improve safety. The objectives were to investigate what policies exist, identify the methods and any technology used for safety incident reporting and consider the data captured in safety incident reports. It aims to contribute to discussions regarding developing systems-based approaches to safety management in care homes.

**Methods:**

A qualitative documentary analysis of safety incident reporting policies in residential and nursing care homes in England was undertaken. Policies were collected from 23 organisations whose staff participated in interviews (n = 75) regarding safety incident reporting between January 2021 until June 2022 and from a structured internet search using specified search terms between April 2022 and May 2022. To be included, a policy needed to refer to safety incident reporting in any capacity and be partially or wholly related to care homes or nursing homes in England. Safety incidents could include staff, residents, contractors, and visitors to the home. Data, extracted using a bespoke framework based on study objectives, were tabulated and analysed deductively and inductively. For the selected policies, the Care Quality Commission website was searched for the latest inspection report and the overall rating was extracted.

**Results:**

Forty-one policy documents were retrieved and screened for inclusion. Twenty-five policies (from 23 organisations) were reviewed. Three were from the internet search and 22 were obtained from interview participants. There was considerable variability in the length and comprehensiveness of the policies, with some homes using untailored, ‘off-the-shelf’ standardised policies produced by a specialist company. Twenty-two (88%) referred to other policy and legislative documents important to safety incident reporting and all but three (12%) policies identified a designated person or role with responsibility for the reports. Only one policy incorporated resident accounts and views into the incident report. Two policies referred exclusively to electronic recording systems with most (n = 19) referring to paper-based reporting systems.

**Conclusions:**

The study identified the extent of, and gaps, in safety incident reporting policies, with reporting practices situated within a broad framework of governance. Incident reporting is as much a matter of governance as practice and there may be a greater opportunity to learn from incident reports than there is currently. Further research about how staff navigate multiple risks, develop adaptive approaches for the contextual conditions, and use safety incident reporting mechanisms within and across care homes to minimise harm may help improve standards, practices and safety in care homes, along with a greater understanding of how policy is utilised in practice.

## Introduction

Care homes (residential care facilities providing temporary or permanent accommodation with personal care and/or nursing care)^1^ in England are legally obliged to provide safe and healthy living and working environments.^2^ Care homes in England provide accommodation, 24-hour personal care such as support with washing, dressing, meals and medication for residents with care needs and frailty who are unable to live independently, or who need respite or palliative care. Additionally, registered nursing homes provide 24-hour in-house nursing care for those with personal and with more complex health needs. Some homes have dual registration, providing both supportive and nursing care. For the purposes of this article, ‘care home’ refers to a residential or nursing home for people with care needs. There are an estimated 14,535 care homes in England.^3^ Between 1 March 2021 to 28 February 2022, English care homes accommodated around 360,792 people.^4^ Care homes are also workplaces for staff and are covered by health and safety legislation for these purposes.^2^ As in health care, safety in care homes involves “the absence of preventable harm and the minimisation of unnecessary risk of harm”^5^(p1).

Care homes are provided by public, private and third sector organisations where different regulatory, commercial and professional governance arrangements apply. All are mandated to provide safe environments for residents and staff^2^ and care homes under contract to the National Health Service (NHS) in England are required to comply with the Patient Safety Incident Response Framework (PSIRF).^6,7^ The PSIRF adopts a systems-based approach, emphasising learning from safety incidents and developing a safety culture to continuously enhance safety and reduce risks^6–8^. Although care homes have similar legal, commercial and reputational risk considerations as healthcare providers, the systems-based approach of the PSIRF does not appear to be applied consistently to safety reporting in care homes. We sought to improve understanding of safety incident reporting in care homes and identify opportunities for sector learning to improve safety, including the potential for a systems-based approach.

## Safety in care homes

Typically, safety management systems foster conditions where the risks of accidents and injuries causing harm are low.^9^ Conceptual approaches to safety management are characterised in Safety-I, Safety-II and Safety-III approaches although debate exists regarding distinctions between them. Safety-I refers to minimising risks of harm through learning from mistakes, Safety-II advocates learning from adaptability in unpredictable circumstances and from resilience to promote safe operations.^9^ Safety III (like Safety-II), represents a systems-based approach to safety and risk management, incorporating risk reduction and learning.^10^

Governance-driven protocols and procedures are important for setting standards for implementation and contribute to developing a systems-level approach to improving outcomes.^11^ The context and concepts of safety in social care are arguably distinct from the universal, error management, system-based directives connected with health care and the NHS.^12^ Although a more holistic concept of safety that acknowledges preferences and actions of individuals within a regulatory and governance framework has been identified in social care, centralised reporting mechanisms are less well developed than in health care in England.^12^ The fragmented nature of care home provision, and the focus of the regulator (the Care Quality Commission) on inspections rather than on development and improvement, can inhibit networking to improve practice in adult social care.^13^ Providers often work independently, in competition and with more explicit commercial interests, creating conditions where policies and procedures are unlikely to be standardised and situated in a national policy framework like the NHS, restricting opportunities for comparison, learning and development. Limited data is available about safety in adult social care at the aggregate (national and regional) level although the government is working with providers and stakeholders to improve the availability and quality of data collected.^14^

The importance of learning from safety events has been recognised by the NHS. The Learn From Patient Safety Events (LFPSE) service in England facilitates learning through early reporting and incident analysis to prevent recurrence and minimise risk.^7^ It encourages the reporting of all events and incidents, even those not resulting in harm, enabling the recording of and access to data about patient safety incidents.^6,7^ The PSIRF aims to improve patient safety and learning from incidents in the NHS and organisations under direct contract (including some care homes).^6^ However, focusing solely on past harms as a mechanism to improve patient safety in the NHS has been challenged and identified as being too narrow an approach,^15^ a criticism also potentially applicable to care homes.

Care homes are complex environments, simultaneously providing homes for residents and workplaces for staff.^2^ In England, the legislative framework for safety in care homes therefore includes the Health and Safety at Work Act 1974 and the Management of Health and Safety at Work Regulations 1999^2^ and other workplace regulations e.g., Manual Handling Operations Regulations 1992 and Control of Substances Hazardous to Health Regulations 2002.^2^ The Reporting of Injures, Diseases and Dangerous Occurrences Regulations 2013 (RIDDOR) places a legal requirement on care homes to report the most serious incidents and diseases to the Health and Safety Executive (HSE). Care home staff record RIDDOR reportable and non-reportable incidents, accidents, and near-misses in care homes. Additionally, there is a legal requirement (under Regulation 18) to report qualifying incidents that result in harm to the Care Quality Commission (CQC),^16^ the independent regulator of health and social care in England. Further obligations apply for the monitoring and reporting of incidents that could lead to abuse or neglect via the Care Act (2014) and other adult safeguarding processes, although these place a different emphasis on risk to safety management approaches.^12^

Statutory and regulatory reporting requirements suggest the dominant approach to safety management corresponds with Safety-I, focusing on errors, learning and risk minimisation to prevent recurrence. Regulators do not ask care homes to report on what has gone well, so they are unlikely to investigate or report instances from which to learn.^9^

## Care home policy and governance

Care home policies are those which align with the definition: “A set of ideas or a plan of what to do in particular situations that has been agreed to officially by a group of people, a business organization (sic), a government, or a political party”.^17^ To fulfil statutory requirements, care homes have policies on incident and accident reporting. This study considers the safety incident reporting policies reviewed, how governance is reflected in the safety incident reporting policy, the messages that these policies conveyed and the extent that they were sensitive to wider discussions around patient safety and incident reporting.

Governance, an ambiguous construct, is compounded by its correspondence with related concepts such as leadership and stewardship and the various frameworks and methods that are used to define and measure these.^18,19^ This imprecision means that it may incorporate policy, regulation, accountability, quality or organisational culture.^18^ Greer’s^19^ useful explanation of governance suggests it embraces both “normative” and “empirical” explanations (p2). For Greer, normative governance refers to expectations regarding standards of how governance *should* operate, so incorporates the potential for measurement and value judgements regarding effective or dysfunctional governance, whereas empirical governance is concerned with *how* governance is practiced, such as how decisions, accountabilities and responsibility operate and policy in practice.^19,20^The empirical element of our analysis involved considering what the policies covered and the normative analysis, a review (value judgement) regarding what should be included in policies to promote and improve safety deriving from incident reporting in care homes – an area that has received little attention so far.

We aimed to develop a better understanding of incident reporting in care homes in England and the potential for learning to improve safety by investigating the research question: What do care home policies say about reporting and recording safety incidents? The policies reviewed outlined the organisations’ instructions regarding actions in the event of safety incidents occurring. In examining safety incident reporting policies, the study contributes to developing an understanding of policies and practices that will be of interest to providers in England, the constituent countries in the United Kingdom and internationally where vulnerable people are cared for by staff in residential care facilities. It also aims to contribute to wider discussions regarding systems-based approaches to safety management and to wider organisational learning across the adult social care sector, converging with the aims of the NHS PSIRF.

## Methods

We conducted: (1) a systematic search and documentary review of care home policies regarding safety incident reporting that were obtained from (a) a systematic Google search and (b) from 75 care home staff who participated in interviews from a nested arm of this study and who consented to share their policies^1^ and (2) a search of the CQC website for the latest inspection report and the overall care home rating (an assessment of the provider’s compliance with regulations and for the delivery of safe, effective, compassionate and responsive care) for the organisations whose policies were reviewed. The 75 interview participants represented residential care (n = 28,), nursing care (n = 12), a combination of residential and nursing care (n = 26) or residential with additional dementia support (n = 9). Job roles included manager (n = 52) and senior roles (n = 23) including chief executive, director (e.g. of operations or compliance) and owner, with many reporting multiple roles. The number of care homes within the participants’ organisations ranged from 1 to 200 (mean = 45, standard deviation = 59). Organisations covered local (n = 37), regional (n = 15) or national (n = 23) geographic areas. Of the 75 interview participants, all (100%) stated they had a policy (43 as a single specific policy and 32 as part of multiple, non-specific policies). Fifty-four (72%) indicated they could share the policy although relatively few (n = 20) did when requested. To maintain anonymity of interview participants (and organisations), quotations from the CQC inspection reports are not provided as they are available online and could be used to identify individual participants.

**Figure 1.**
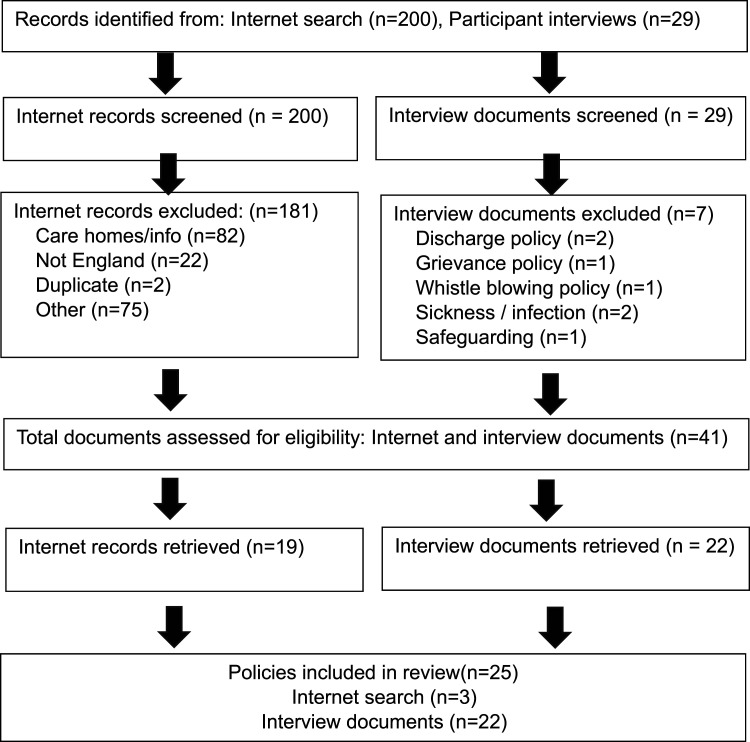
Retrieval and screening.

Altogether, 25 policies (22 policies from 20 care home interviews as two care homes had more than one policy included in the review) and 3 from the Google search) met the inclusion criteria and were reviewed (Figure 1). Regarding the type of care home the policies (n = 25) covered, 12 were care homes with dual registration, providing residential and nursing home care, 8 were residential care, 4 were nursing, and 1 was not known.

We focused on aspects influenced by governance, exploring what policies existed, and as outlined in the following Data Extraction and Data Analysis sections, assessed comprehensiveness and scope. Given the legislative framework for care home regulation and reporting and the links to related policies (e.g. safeguarding) the analysis was situated in the wider policy landscape and captured in an Excel spreadsheet. Ethical approvals were obtained from the Health Research Authority (reference: 286904)) and Northumbria University (reference 120.2450).

### Data sources and inclusion criteria

As this policy review was part of a wider study^1^ policies were included from the interview participants who shared their policies and from the Google search if they referred to safety incident reporting in any capacity from or related (partially or wholly) to care homes or nursing homes in England. An additional manual search was undertaken on the CQC website to identify the specific care homes whose incident reporting policies were reviewed in this study. CQC reports were reviewed for their overall assessment and for references to incident reporting during inspection and considered in relation to the qualitative documentary analysis of their policies.

Two Google searches identified care home policy on incident reporting (search 1, 22^nd^ April 2022), and the identification of commissioner and regulatory policies on care home incident reporting policy (search 2, 16 May 2022). The following search terms and Boolean logic were used:

**Search 1:** (“care home*” OR “nursing home*” or “residential home”) AND (“policy” OR “guideline*” OR “guidance” OR “strategy” OR “protocol”) AND (“incident report” OR “error report” OR “adverse event” OR “accident report” OR “falls” OR “medication” OR “aggression”)

**Search 2:** (“care home*” OR “nursing home*” or “residential home”) AND (“local authority” OR “Clinical Commissioning Group” OR “Care Quality Commission” OR “council”) AND (“policy” OR “guideline*” OR “guidance” OR “strategy” OR “protocol”) AND (“incident report” OR “error report” OR “adverse event” OR “accident report” OR “falls” OR “medication” OR “aggression”)

As indicated, care homes policies were sourced from two routes, 1) Google Search and 2), from interview participants in the scoping study. Following recommended guidance for evidence reviews using Google^21^ the first 100 hits for each search term were retrieved and copied into a Microsoft Word document. Interview data obtained from participants from the wider study examining safety incident reporting in care homes^1^ occurred January 2021 until June 2022. All (75) participants were asked if their home had a policy on incident reporting and whether they would share the policy with the research team. Care home policies provided were emailed to the research team. A summary of retrieval and screening is provided in Figure 1.

### Screening

Policies were screened using a two-step approach. First, MS screened the title or url link and scope against the inclusion criteria. Online documents were obtained from the Google link, and inclusion and exclusion criteria applied to check eligibility. Second, twenty percent of the retained results were double screened by KS. No disagreements regarding eligibility were identified. Altogether, 229 documents were screened (200 from the Google search and 29 from interview participants) and 188 documents were excluded (181 from the internet search and seven from the internet search). Screened documents were read by MS.

### Data extraction

The following data items were extracted from 25 policies from 23 care homes included in the dataset: incident report completion, the process(es) for sharing incident reports, technologies used, policy date, who it was aimed at, reporting responsibilities, who received or reviewed incidents and whether consistent procedures applied for all incident reporting. Data were extracted by MS, 10% were checked by JS and queries or disagreements resolved between the two authors. Each policy was given an identifier, and data tabulated using Microsoft Excel (see Table 1 for summary data).

**Table 1.** Review of policies.

ID	Service: nursing (Nurs), residential (Res), mixed (Mix), other (Oth)	Review interval/period	Local (L), Regional (R), National (N)	Incident type specified	No. of homes in organisation	No. of beds/rooms in care home	States type of data collected	Recording method	Post-incident monitori/investigation	Links with other policies or screening tools	Post-incident learning	Ownership - person/role specified	Creates/suggests accounta-bility/blame	Resident involved	Duty of candour/being open?	Overall CQC rating (aggregated rating based on the 5 key questions)
1	Mix	Yes		Falls	Crisis	N/A	Yes	Paper	Yes	Yes	No	No	No	No	No	N/A
2^a^	Nurs	Yes	N	Non-resident accidents, near misses, dangerous occurrences	100	21-40	No	Paper	Yes	Yes	Yes	No	No	No	No	Good
3^a^	Nurs	No	Nat	Accidents, near misses, behaviour causing harm/risk, restraints and restrictive practices, medication errors	100	21-40	No	Paper	Yes	Yes	No	No	No	No	No	Good
4	Res	No	Reg	Accidents, injuries	4	21-40	Yes	Electronic	Yes	Yes	Yes	Yes	Yes	No	Yes	Good
5	Nurs	Yes	Loc	Accidents, incidents, near misses	2	21-40	Yes	Paper	Yes	Yes	Yes	Yes	Yes	No	Yes	Good
6	Res	Yes	Nat	Accidents, incidents, near misses, injuries	13	21-40	Yes	Paper	Yes	Yes	Yes	Yes	Yes	No	Yes	Good
7	Mix	Yes	Loc	Accidents, incidents and ill health	2	41 or over	Yes	Both	Yes	Yes	Yes	Yes	No	No	No	Good
8	Mix	Yes	Reg	Incidents	2	41 or over	Yes	Electronic	Yes	No	Yes	Yes	Yes	No	No	Good
9	Mix	No	Reg	Accident, incident, injury, deaths, injuries, violence, dangerous occurrences	7	41 or over	Yes	Paper	Yes	Yes	Yes	Yes	Yes	No	Yes	Good
10	Mix	No	Nat	Accidents or injuries, falls, observed, allegations	82	41 or over	Yes	Paper	Yes	Yes	Yes	Yes	Yes	No	Yes	Good
11	Mix	No	Nat	All accidents, injuries, incidents, near misses, dangerous occurrences	1	21-40	No	Paper	Yes	Yes	Yes	Yes	Yes	No	No	Good
12	Mix	Yes	Nat	Accidents, incidents, near misses	6	20 or under	No	Electronic	Yes	Yes	Yes	Yes	Yes	No	Yes	N/A*
13	Mix	Yes	Reg	Incidents, accidents, near misses	6	41 or over	Yes	Both	Yes	Yes	Yes	Yes	Yes	No	Yes	Good
14	Res	Yes	Loc	Accidents, injuries, near misses	1	21-40	Yes	Paper	Yes	Yes	Yes	Yes	Yes	No	Yes	Good
15	Nurs	No	Loc	Accidents, injuries	1	41 or over	No	Paper	Yes	Yes	Yes	Yes	Yes	No	Yes	Good
16	Res	Yes	Loc	Accident, near misses, dangerous occurrences	1	41 or over	Yes	Paper	Yes	Yes	Yes	Yes	Yes	No	Yes	Requires improvement
17	Mix	Yes	Reg	Accidents, incidents, near misses	6	41 or over	Yes	Paper	Yes	Yes	Yes	Yes	Yes	No	Yes	N/A*
18^b^	Res	Yes	Reg	Accidents, injuries, near misses	10	20 or under	Yes	Paper	Yes	Yes	Yes	Yes	Yes	No	Yes	Good
19^b^	Res	Yes	Reg	Serious incidents	10	20 or under	No	Paper	Yes	Yes	Yes	Yes	Yes	No	Yes	Good
20	Mix	Yes	Nat	Accidents, incidents, near misses	1	20 or under	No	Paper	Yes	Yes	Yes	Yes	Yes	No	Yes	Good
21	Res	Yes	Loc	Accidents, incidents, near misses	3	20 or under	Yes	Paper	Yes	Yes	Yes	Yes	Yes	No	Yes	Good
22	Res	No	Reg	Minor, moderate or serious incidents	125	620 or under	No	Electronic	Yes	No	Yes	Yes	No	No	No	Good
23	Not known	No	Yes	Medication errors	Routine	Not known	No	Paper	Yes	No	No	Yes	No	No	No	Good
24	Mix	Yes	Yes	Falls related to warfarin overdose or bleeding, medication errors	Routine	N/A (LA policy)	Yes	Paper	Yes	Yes	No	Yes	Yes	Yes	No	N/A*
25	Mix	Yes	Nat	Incident reporting for falls	10	41 or over	Yes	Paper	No	Yes	No	Yes	No	No	No	Good

NA* Policy is for a Trust, local authority or organisation so not attributed to an individual care home.

^a^These policies were from the same establishment.

^b^These policies were from the same establishment.

The CQC website was searched for the care home which the selected policies related to, and the overall rating from the latest inspection report was extracted and included in the table. Although the policy was the unit of analysis, linking our review with the latest CQC report was useful to gain an overall assessment of how well the care home was performing. The CQC’s inspection regime focuses on five equally weighted key questions: “Are they safe?”, “Are they well-led?”, “Are they effective?”, “Are they caring?” and “Are they responsive?”^22^ The CQC provides individual ratings for the five key questions and an overall rating (drawn from the ratings for the five key questions) is then given to the care home.^22^

### Data analysis

Using the framework approach to data analysis,^23^ data were categorised and organised in tabular form in an Excel spreadsheet inductively and deductively.^24^

Research aims and objectives pertaining to the policy review included to “Investigate, using desk-based approaches, what policies exist for incident reporting, the technology used to incident report, and the types of data captured within incident reports”^1, (p.2.)^. Processes for policy retrieval and agreed topics of interest for data extraction and analysis were outlined in our research protocol. Types of data captured, the technology used to record the incident and policy dates/review periods are examples of the deductive data coded. Detailed reading of the policies further allowed for the extraction of content to inform an inductive analysis and to generate previously unanticipated insights related to the understanding of safety incident reporting policies. During project team meetings, key topics for analysis were discussed and agreed. Policies were read multiple times and key phrases and topics informed the systematic and iterative development of the framework that was then applied to all policies. The tabulating process included reflecting on the style and articulation of policies and how easy they were to follow. The development of the framework was informed by Greer’s concepts of normative (the policies in place) and empirical governance (the activity, administration and accountabilities included in the policies) such as what the policies were and their scope.^19^

## Results

This section focuses on empirical governance (policy content). Twenty-five policies were included in the study with document length ranging from 1 to 129 pages (mean = 12 pages, median = 7 pages, mode = 13 pages).

### Review periods and safety incident recording method

Few incident reporting policies were readily available online. All policies (n = 25) stated the policy aims and stipulated that any incident (the what, how and why) be recorded. Most (n = 19) used a paper recording method, while two used electronic methods and four used both paper and electronic methods. A policy summary is presented in Table 1. The majority (n = 21) applied to specific care homes not the wider organisation or care group. Most (n = 18) had policy or review dates specified, ranging from 2008 to 2022. Seven were undated, and eight had no policy review date. The remaining policies either included a review date, indicated a periodic review or that review was undertaken when required. Six care homes used the same incident reporting policies drafted by a specialist company with the establishment’s name updated and the contents of the policy replicated.

### Process

Variation in policy length, to some degree, reflected whether other policy domains were included and whether it was focused solely on a care home, care home provider or signposted other organisational documents. The policy with 129 pages was aimed at residential and day care facilities and staff where the local authority was responsible for service delivery. It included guidance on medication, controlled drugs and the use of oxygen. Another policy with three pages included accidents, incidents and the procedure for staff to report sickness and incapacity for work.

Most (n = 24) policies were routine (not developed in response to a crisis), relating to incident and serious incident reporting, except one policy that was a joint falls protocol. All policies (n = 25) included policy aims and typically covered: accidents, incidents, dangerous occurrences, near misses, injuries, medication errors and falls on the premises (or externally if they related to care provision)

Although the following definition from a reviewed policy appears straightforward, the absence of precision could mean that some events are at risk of not being recorded (such as acts of omission or events that are considered normal such as falls) and of focusing more on novelty not harm or the potential for harm:

“This care service describes an “incident” as an unexpected event, something that occurs in passing or out of the ordinary course of events. It accepts that many incidents are unremarkable and are not concerning, but some are, and they need to be reported and recorded by the care staff involved.” (Policy 3)

For simplicity, in this paper the term ‘incidents’ is used to incorporate all the events resulting in harm or potential harm.

### Coverage

Less commonly, some policies incorporated the reporting of allegations, violence, behaviour causing harm or risk of harm and the use of restrictive practices. This was important because their inclusion demonstrated a wide definition of incidents incorporating any with the potential to create harm. Policies analysed covered residents, staff, visitors, visiting professionals, contractors, volunteers, stakeholders and members of the public:

“This policy applies to all [organisation’s] services, External Affairs, central teams and activities, staff, contractors, volunteers, service users and members of the public visiting [organisation’s] workplaces or service settings.” (Policy 19).

Two policies identified external events connected with care home activities, one mentioned incidents regarding falls only and another’s focus was on medicine administration and incident reporting regarding medication errors. Thirteen policies contained at least one reference to safeguarding:

“If there is an injury, appropriate first aid/medical attention must be sort [sic] and the appropriate people informed including NOK [Next of Kin]. This may include safeguarding if there is concern as to how the injury occurred.” (Policy 8).

“A 999 ambulance should always be considered if…There are any safeguarding concerns (e.g. possible non–accidental injury or a vulnerable person is affected)… If necessary, Safeguarding should be informed and if required the Police.” (Policy 10)

“Care staff should report and record any alleged, suspected or actual abuse of a service user in line with the service’s safeguarding policy and procedures so that the appropriate safeguarding actions can be taken.” (Policy 3)

One policy acknowledged safeguarding applying to staff, not residents:

“…This is necessary so that the Health and Safety Executive HSE can determine trends and patterns in workplace accidents and put in place legislation and guidelines that will safeguard workers all over the UK.” (Policy 9)

### Regulation and reporting

All 25 policies included references to post-incident monitoring or investigation and almost all (n = 22) referenced or included links to other policies or screening tools which were either internal to the care home or organisation or were national policies. Typically, links to external policy references included the Reporting Accidents and Incidents at Work (RIDDOR, 2013), Health and Safety legislation, the CQC, the Health and Social Care Act, Mental Capacity Act, the Medicines and Healthcare Products Regulatory Agency, Duty of Candour legislation and the National Institute for Health and Care Excellence (NICE). Examples of internal documents related to First Aid, Risk Assessments, Safeguarding, Serious Incident Reporting and Accident Reporting policies.

Twenty of the 25 policies specifically referred to post-incident learning to minimise recurrence and only three omitted including a named person or job role with allocated responsibility for the incident report. Five did not explicitly state post incident learning. These included: Policy 23 (medication errors), Policy 24 (which covered all the local authority residential and day care services it operated, incorporating procedures for reporting and recording and referring to other supporting documents and policies) and Policy 25 (a falls prevention policy). The fourth (Policy 1), was a falls protocol developed for use across a county, referring to other screening tools. The fifth (Policy 3) was a serious incident reporting policy for the care group that signposted other internal documents.

### Participation and transparency

Only one (Policy 24) incorporated resident accounts and views into the incident report, although these accounts and views may not have been appropriate for all reporting topics, such as staff accidents. This policy was in the context of self-administration of medication for maintaining independence. Post-incident support was stipulated in one organisation’s Serious Incident Notification Policy and Procedure, also notable because it involved a consideration of emotional and psychological harm as well as physical that was absent in all other policies:

“…all person(s) involved in the incident all reasonable support necessary to help them overcome the physical, psychological and emotional impact of the incident.” (Policy 19)

Over half (n = 14) of the policies referred to a duty of candour. The duty of candour (statutory and professional) legislation compels providers and staff to openness and transparency with people who use their services and regarding mistakes or safety incidents.^25^ One policy that did not refer to a duty of candour indicated staff involved with the incident:

“Aside from informing the duty manager and making a report, staff must maintain strict confidentiality relating to the details of any accident or incident that occurs on the premises, or involves staff, volunteers, residents or visitors to the care home.” (Policy 5)

Apart from one care home, all those eligible for inspection were assessed by the CQC as having good overall performance at their last inspection. The one exception, (Policy 16, relating to one care home) was rated as requiring improvement including on the questions regarding safety and being well-led. Written feedback in the CQC inspection report for this care home identified a large amount of accident and incident reports at the establishment. The inspection report acknowledged that detailed information was recorded, but analysis to identify issues and patterns to learn from and prevent recurrence was absent. Similarly, the care home with Policy 4 was rated as good overall but rated as requiring improvement regarding the key question of being well-led and included a recommendation to revise accident and incident monitoring.

The CQC inspection reports we reviewed noted positive actions by providers regarding incident reporting and monitoring for service improvement. For example, CQC inspection comments acknowledged that systems were in place to facilitate learning for 8 care homes (Policies 2 and 3 were from the same organisation) and six of these policies applied across the care group that owned the care home). Of the providers whose policies were reviewed as part of this study (Policy 5, Policy 10, Policy 13, Policies 2 &3, Policy 23, Policy 25), an open culture operated (Policy 9), and appropriate recording and analysis to minimise risks of recurrence was routine (Policy 6).

## Discussion

Our analysis of care home policies showed the empirical governance of safety in terms of administration, coverage, regulation and reporting, participation and transparency. Here, we compare these findings with safety incident reporting as a systems-based approach to offer normative concept(s) of governance for care homes and consider what could be included based on approaches that have been applied to health care.

### Governance: Compliance versus learning

CQC ratings for organisational safety assess leadership, management and governance that promotes learning, minimises avoidable harm, and develops staff. These aspects are related to the overlapping purposes of governance identified by Greer^19^ and resonate with the Safety-I (learning from errors) and Safety-II (learning from what has gone right) approaches. Incident reporting policies reviewed in this study suggest that care homes are predominantly taking a Safety-I approach to risk management approach and learning from near misses and mistakes while complying with statutory reporting requirements. Utilising Greer’s^19^ definitions, empirical governance (governance activity) was demonstrated by the care homes represented in the study through the safety incident reporting policies and the specification of reporting mechanisms and accountability. However, empirical governance alone (in the form of policies for reporting and recording safety incidents) does not determine safety, as the CQC inspection rating for the care home rated overall as requiring improvement indicated. Incident reporting is associated with safety management. The small number of CQC inspection reports we considered as part of this review had a standardised heading for CQC reflection on incident reporting in the ‘Learning lessons when things go wrong’ section of the report. As indicated, learning from what has gone well is not an explicit part of the CQC’s remit.^9^ However, including a heading for ‘Learning when things go well’ could facilitate learning and sharing practice within the sector. Reflecting on what has gone well in risky, unstable and complex environments could create an important avenue for learning in care home settings, moving beyond compliance with legislation to sector-wide learning.

### Systems-based approaches to safety

Normative governance, what could or should be achieved, of care homes in relation to safety incident reporting, could incorporate learning from the systems-based approach to safety used in health care. Safety culture is situated as concentrating on incidents as a systems-based response.^6,8^ Through this lens, care home policies could be judged in terms of their comprehensiveness and potential to facilitate learning to improve safety incident reporting and management. As noted, a variety of legislative and institutional obligations exist regarding safety incident reporting in care homes. Normative governance would establish balance between compliance and learning, proactivity regarding resident (and staff) wellbeing, safety and reputational risk.

The specific setting of care homes, where residents with increasingly complex and multiple health needs are cared for^9^ by a workforce experiencing recruitment and retention difficulties, create challenging and complex environments. Smith et al.’s^26^ study of Safeguarding Adult Reviews in care homes for older adults identified five domains for safety: harm, reliability, sensitivity to operations, anticipation and preparedness, integration and learning. The study provides a useful framework for care home settings to consider, promoting adaptable and appropriate responses and contributing to embedding a safety culture in practice.^26^ Recognising the severity of an incident (such as a fall, which might be unavoidable for some residents^26^), conveying information on staff changeover and reviewing care plans can prevent further harm.^26^ Good leadership, management, staff training and time for engagement in improvement planning are also important.^26^ This implies an approach to safety, drawing on Safety-I and Safety-II, that goes beyond incident reporting.

### Standardisation, transparency, resident engagement and psychological harm

The questions of standardisation, transparency, resident engagement and psychological harm should also be considered to further align with systems-based approaches to safety and are of potential relevance to aspects of normative governance of care homes in relation to safety incident reporting.

Using ‘off-the-shelf’ policies may create the opportunity to standardise policies across care homes, which should their implementation also be standardised, may result in better information quality for drawing comparisons between different care home providers and facilitate improved possibilities for learning. The scope and emphasis of the policies reviewed varied, yet similar aims and purpose were evident, reflecting sector-wide legislative, and consequently institutional, drivers. Shared dimensions in policy development may, for example, include whether a tight or loose definition of safety incidents was used, whether the policy focused on harm or non-harm, its inclusivity (residents) or exclusivity (staff and visitors). This suggests there is the potential to develop a typology of dimensions - a framework - for safety incident reporting policies within the care sector. This could generate an opportunity for standardisation as a mechanism to raise standards, improve safety, transparency, promote learning and represent a shift towards a systems-based approach to safety incident reporting and management despite the diversity of care home providers and provision. It may aid joint working within and across sectors (e.g. with the NHS). Relatedly, electronic record keeping may enable operational staff and managers to access safety incident reports more easily, enhancing opportunity for learning and risk identification within and across organisations. Electronic record keeping also provides scope to use artificial intelligence as an additional avenue for learning, yet few organisations in this review recorded safety incident reports electronically. Delivering ongoing public services, however, may mean that approaches vary across providers and links to service users and wider policy and statutory requirements are necessary, and relatedly, accountability and monitoring are required.^20^ Furthermore, contract specifications may not be standardised across local authorities and different contract specifications may be issued to providers,^20^ adding complexity, especially as care homes provide personalised services to their residents and larger providers may have care homes spanning local authority areas. This is relevant for Patient Safety Incident Response Framework implementation in care homes, which although written for NHS organisations, has repercussions for other care settings with the requirement to make policies publicly available.

Publicly available policies could be important for developing trust with residents and their family members to demonstrate that robust and transparent policies exist. Trust between organisations and staff is core for developing a just culture^27^ and this should also extend to residents and family members. Greater transparency could also be achieved by incorporating residents’ perspectives on the safety and quality of their care. Speaking to residents to obtain their views is linked with improving safety and learning from incident reporting^19^ and minimising risks of recurrence.^28^ Our analysis, albeit on a limited sample, demonstrated that only one care home collected resident narratives of incidents. This suggests this data is not routinely collected. However, it is arguably contrary to the spirit of openness and duty of candour, and represents a gap for organisational learning that care home providers and regulators could consider addressing.^26^

Safety incident reporting policies covering resident harm focused on recording physical harms, with only one serious incident policy referring to post-incident (physical, psychological and emotional) support. However, a wider definition of adverse events includes psychological and social as well as physical harm.^28^ Only recording physical harms may be deficient,^15^ particularly in the context of care homes where psychological harm has an increased relative importance because care homes are resident’s homes as well as workplaces.^26^ Recording psychological harm potentially provides insights for harm avoidance in diverse and volatile environments. Incident policies should include clear references to other closely linked policies such as safeguarding (which typically include references to psychological, physical and sexual harm and abuse), to medicines management and falls prevention policies to ensure that a comprehensive safety system exists.

The empirical governance of safety incident reporting in care homes demonstrates that incident reporting is recognised as a quality of care mechanism to improve safety.^11^ Incident reporting policies and implementation in health care reside within the governance framework associated with quality of care, under the umbrella of improving clinical practice.^11^ The normative governance of care home safety incident reporting, in comparison with safety systems, could enhance the reporting and learning by providers and regulators in a way that is both sensitive to the operating environment and considers risks as well as breaches to policy.^15^ We are not able to assess from our study whether any omissions we identified were due to a perfunctory approach with little or no engagement with the contexts of the care homes and residents, or if they reflected practical and operational issues regarding staffing availability and specialism to draft policy.

## Limitations

A relatively small number of policies (obtained from interview participants or accessed through internet searching) were reviewed and did not provide a representative sample of care home safety incident reporting policies. Further, the sample obtained from interview participants willing to share their policies may have reflected care homes with an embedded safety culture. Future research could specifically focus on policies from homes that have been rated by the regulator as requiring improvement or are failing, though these may be more difficult to identify and access.

## Conclusion

The study identified the comprehensiveness of incident reporting policies and situated practices within a broad framework of governance. Incident reporting is as much a matter of governance as practice and there may be a greater opportunity to learn from incident reports than there is currently. The transparency of care homes regarding risks to safety, the management of risks and whether the function of regulation is enforcement or assistance are important considerations and may affect how open organisations are regarding approaches to safety management^29^ and incident reporting. This has implications for developing safety incident reporting systems and sharing learning. The development of electronic systems for reporting and learning are likely to improve safety incident reporting and these aspects are situated within the sphere of the governance of safety management. Incorporating a consideration of how organisations demonstrate achievement of these as part of statutory reporting mechanisms may support the development of conditions for improving safety.^15^

Recording incidents, identifying risks to safety and how safety and care can be improved in care homes remain important. Incident reporting may contribute to demonstrating the fulfilment of regulators’ and commissioners’ requirements regarding being safe and well-led, aspects relevant to providers nationally and in other countries with similar provision and care home resident profiles. Future research could examine how incident reporting policy is used in day-to-day practice, as well as how the policy is generated and actioned by policy-makers, commissioners, regulators, care staff and, specific to the context of care homes, the role of residents and family members. This should incorporate reporting processes and investigation phases and identify contributions to organisational and sector-wide learning.

## Data Availability

The participants sharing their policies did not consent for their data to be shared publicly and sharing the publicly available data we accessed could lead to the identification of care home organisations so supporting data are not available.

## References

[1] Scott J Brittain K Byrnes K , et al. Residents transitioning between hospital and care homes: protocol for codesigning a systems-level response to safety issues (SafeST study). BMJ Open 2022; 12: e050665. 10.1136/bmjopen-2021-050665 PMC8739053 34992105

[2] Health and Safety Executive . Health and safety in care homes. 2nd ed. Health and Safety Executive, 2014.

[3] Munston S . Care home stats: number of settings, population and workforce. carehome.co.uk, 2022.

[4] Barrett S . Care homes and estimating the self-funding population, England: 2021 to 2022. ONS, 2022.

[5] Rand S Smith N Jones K , et al. Measuring safety in older adult care homes: a scoping review of the international literature. BMJ Open 2021; 11: e043206. 10.1136/bmjopen-2020-043206 PMC7957135 33707269

[6] NHS England . Patient safety incident response framework. NHS England, 2024.

[7] NHS England . Patient safety incidence response framework, version 1, August 2022. Report. NHS England, 2022.

[8] NHS England . The NHS patient safety strategy: safer culture, safer systems, safer patients. NHS England, 2019.

[9] Hollnagel E Wears R Braithwaite J . From Safety-I to Safety-II@ A white paper. From Safety-I to Safety-II: a white paper. In: The resilient health care net: published simultaneously by the University of Southern Denmark. Macquarie University, Australia, 2015.

[10] Aven T . A risk science perspective on the discussion concerning Safety I, Safety II and safety III. Reliab Eng Syst Saf 2022; 217: 108077.

[11] Tello JE Barbazza E Waddell K . Review of 128 quality of care mechanisms: a framework and mapping for health system stewards. Health Policy 2020; 124: 12–24. 31791717 10.1016/j.healthpol.2019.11.006 PMC6946442

[12] Scott J Birks Y Aspinal F , et al. Integrating safety concepts in health and social care. J Integrated Care 2017; 25: 76–83.

[13] Hudson B . Clients, consumers or citizens? The privatisation of adult social care in England. Policy Press, 2021.

[14] Department of Health and Social Care . Guidance: care data matters: a roadmap for better adult social care data. DHSC, 2023. Report.

[15] The Health Foundation . A framework for measuring and monitoring safety: a practical guide to using a new framework for meassuring and monitoring safety in the NHS. Report. The Health Foundation, 2016.

[16] Care Quality Commission . Regulation 18: notification of other incidents. Care Quality Commission, 2023.

[17] Cambridge Dictionary. Meaning of policy in English. Avaliable at: https://dictionary.cambridge.org/dictionary/english/policy (Accessed: 30/6/23).

[18] Barbazza E Tello JE . A review of health governance: definitions, dimensions and tools to govern. Health Policy 2014; 116: 1–11. 24485914 10.1016/j.healthpol.2014.01.007

[19] Greer S . Organization and governance: stewardship and governance in health systems. In: van Ginneken E Busse R (eds). Health care systems and policies. Springer Link, 2018.

[20] Spicker P . Social policy: theory and practice. 3rd ed. Policy Press, 2014.

[21] Haddaway NR Collins AM Coughlin D , et al. The role of Google scholar in evidence reviews and its applicability to grey literature searching. PLoS One 2015; 10: e0138237. 26379270 10.1371/journal.pone.0138237 PMC4574933

[22] Care Quality Commission . How CQC monitors, inspects and regulates adult social care services: November 2021. Report. Care Quality Commission, 2021.

[23] Ritchie J Spencer L O’Connor W . Carrying out qualitative analysis. In: Ritchie J Lewis J (eds) Qualitative research practice. London: Sage, 2009.

[24] Leavy P . Research design: quantitative, qualitative and mixed methods, arts-based and community-based participatory research approaches. The Guildford Press, 2023.

[25] Care Quality Commission . Regulation 20: duty of candour. Report. Care Quality Commission, 2022.

[26] Smith N Rand S Morgan S , et al. The scope of safety in English older adult care homes: a qualitative analysis of safeguarding adult reviews. J Adult Prot 2023; 25: 3–13.

[27] Paradiso L Sweeney N . Just culture: it’s more than just a policy. Nurs Manag 2019; 50: 39. 10.1097/01.NUMA.0000558482.07815.ae PMC6716556 31094886

[28] St Clair B Jorgensen M Nguyen A , et al. A scoping review of adverse incidents research in aged care homes: learnings, gaps, and challenges. Gerontol Geriatr Med 2022; 8: 23337214221144192. 36568485 10.1177/23337214221144192 PMC9772958

[29] Illingworth J . Developing and testing a framework to measure and monitor safety in health care. Clin Risk 2014; 20: 64–68. 25419166 10.1177/1356262214535735 PMC4230959

